# TAp63α Is Involved in Tobacco Smoke-Induced Lung Cancer EMT and the Anti-cancer Activity of Curcumin via miR-19 Transcriptional Suppression

**DOI:** 10.3389/fcell.2021.645402

**Published:** 2021-03-04

**Authors:** Chunfeng Xie, Jianyun Zhu, Xue Yang, Cong Huang, Liping Zhou, Zili Meng, Xiaoting Li, Caiyun Zhong

**Affiliations:** ^1^Department of Nutrition and Food Safety, School of Public Health, Nanjing Medical University, Nanjing, China; ^2^Department of Digestive Disease and Nutrition Research Center, The Affiliated Suzhou Hospital of Nanjing Medical University, Suzhou, China; ^3^Department of Clinical Nutrition, Nanjing Drum Tower Hospital, Nanjing, China; ^4^Guangde Center for Diseases Prevention and Control, Guangde, China; ^5^Cell Therapy Center, Xuanwu Hospital Capital Medical University, Beijing, China; ^6^Department of Respiratory Medicine, Huai’an First People’s Hospital, Nanjing Medical University, Huai’an, China; ^7^Center for Global Health, School of Public Health, Nanjing Medical University, Nanjing, China

**Keywords:** TAp63α, lung cancer, miR-19, EMT, curcumin, tobacco smoke

## Abstract

As a key risk factor for lung cancer, tobacco smoke (TS) influences several cellular processes, including epithelial-mesenchymal transition (EMT). TAp63α is a crucial transcription factor involved in tumor progression. The present study was designed to investigate the potential role and underlying mechanisms of TAp63α in TS-induced lung cancer EMT. We found that compared to normal tissues, the tumor tissues collected from lung cancer patients showed a lower level of TAp63α expression, along with downregulated E-cadherin expression and upregulated Vimentin expression. Results of treatment with TAp63α and TAp63α siRNA as well as with tumor growth factor-β (TGF-β) showed that TAp63α acted as a tumor suppressor gene, and its upregulated expression suppressed lung cancer EMT. Significantly, TS exposure altered expression of EMT-related markers, enhanced cell migratory and invasive capacities, and decreased the TAp63α expression level in lung cancer cells. Overexpression of TAp63α significantly alleviated TS-stimulated lung cancer EMT. Mechanistically, TAp63α expression transcriptionally reduced the miR-19 level, which resulted in the suppression of lung cancer EMT. Additionally, as a natural compound possessing anti-cancer effects, curcumin inhibited TS-induced lung cancer EMT by increasing TAp63α expression and reducing miR-19 expression. Collectively, our results indicate that TAp63α inhibits TS-induced lung cancer EMT via transcriptionally suppressing miR-19 and the inhibitory effect of TAp63α on miR-19 mediates the anti-cancer action of curcumin. These findings provide new insights into novel targets for lung cancer prevention.

## Introduction

Tobacco smoke (TS) contains numerous mutagens and carcinogens, and it constitutes a well-known lung cancer risk that causes 90% of lung cancer cases (Lemjabbar-Alaoui et al., 2015). Epithelial-mesenchymal transition (EMT) is a necessary process that drives metastasis during lung cancer development (Zhang et al., 2012; Vu et al., 2016). TS exposure can induce the accumulation of multiple molecular abnormalities and lead to lung cancer occurrence and development (Zhang et al., 2012; Pillai et al., 2015; Vu et al., 2016). The inactivation of specific tumor suppressor genes is also correlated with lung cancer progression (Ji et al., 2007; Lin et al., 2010; Li et al., 2019).

The transcription factor p63 belongs to the p53 family and plays a vital role in cancer pathogenesis. It is transcribed into two isoforms: activating ΔNp63 and inhibitory TAp63 (Moll and Slade, 2004; Bankhead et al., 2020). Each isoform can be spliced into three transactivating domains (α, β, γ), of which α has been demonstrated to be the essential domain in tumorigenesis (Carroll et al., 2006). ΔNp63α exhibits oncogenic action against p53 family members. TAp63 is functionally similar to wild-type p53 regarding its tumor suppressive function. However, the function of TAp63α in tumor progression is tissue/cell type-dependent. Previous studies have shown that TAp63 is downregulated in lung cancer specimens (Lo et al., 2011). TAp63 repression is involved in the promotion of lung cancer EMT by long non-coding RNA SNHG1 (Zhang et al., 2017). However, the possible role and relevant mechanisms of TAp63α in TS-stimulated lung cancer EMT are still poorly understood.

MicroRNAs (miRNAs) are small non-coding RNAs that modulate multiple solid tumor pathology processes (Rupaimoole and Slack, 2017; Ingenito et al., 2019). Among the miR-17∼92 clusters, miR-19 (containing two isoforms: miR-19a and miR-19b) is an essential oncogenic gene in tumorigenesis (Olive et al., 2009). Higher level of miR-19 has been detected in clinical specimens from lung cancer patients (Peng et al., 2018; Qiu et al., 2018). The overexpression of miR-19 promotes lung cancer cell proliferation (Peng et al., 2018). Lin et al. (2014) found that TAp63α inhibited miR-133b to suppress the metastasis capacity of colon cancer. However, the link between TAp63α and miR-19 in lung cancer EMT has not been reported.

Recently, extensive preclinical studies have revealed that curcumin acts as a chemopreventive agent for cancer. Curcumin is a well-known natural polyphenol derived from turmeric (*Curcuma longa*). The effective inhibition of cancer by curcumin is presented through various mechanisms, including the suppression of cell proliferation, migration, and invasion, by targeting numerous genes. Our previous data showed that curcumin alleviated chronic TS exposure-induced urocystic EMT through the Wnt/β-catenin signaling pathway (Liang et al., 2017). Nevertheless, the protective effects of curcumin against TS-triggered lung cancer EMT via regulation of TAp63α are still elusive.

The present study was designed to investigate the role and potential mechanisms of TAp63α on TS-triggered lung cancer EMT, along with the preventive effect of curcumin on TS-induced lung cancer.

## Materials and Methods

### Clinical Samples

Thirty-two specimens of lung cancer tissue and the corresponding relative normal tissues were obtained from Huai’an First People’s Hospital Affiliated with Nanjing Medical University. An experienced pathologist evaluated all tissue sections to confirm the diagnosis of non-small cell lung cancer (NSCLC), according to the WHO classification. The Ethics Committee approved all procedures involving human tumors of Nanjing Medical University (ethical clearance application number 2016-318). Written informed consent was obtained from all patients who participated in this study. The baseline patient characteristics were listed in Table 1. Collected specimens were frozen in liquid nitrogen immediately after surgical resection and maintained at −80°C until protein extraction.

**TABLE 1:** Baseline patient characteristics.

**Characteristic**	**Patients, n**
**Gender**	
Female	5
Male	27
**Age (mean ± SD)**	60.91 ± 8.43
**Smoking history**	
Smoking	21
Non-smoking	11
**Tumor stage**	
I	13
II	9
III	10
Total	32

### Cell Culture and Cigarette Smoke Extract Preparation

Human lung cancer H1299 and A549 cell lines were purchased from the Shanghai Institute of Cell Biology, Chinese Academy of Sciences (Shanghai, China). A549 and H1299 cells were cultured in RPIM 1640 medium supplemented with 10% fetal bovine serum (Gibco, Grand Island, NY, United States), 100 U/mL penicillin, and 100 μg/mL streptomycin (Gibco), and then incubated at 37°C in an atmosphere of 5% CO_2_. TGF-β (purity: ≥98%) was purchased from APExBIO Technology LLC (Houston, United States). Cigarette smoke extract (CSE) was prepared daily, immediately before use, according to a previously reported protocol (Wang J. et al., 2018; Xie et al., 2019).

### Western Blot Analysis

Proteins from cells and tissues were collected using a lysis buffer. The BCA assay kit was used to measure the protein concentration. Immunoblotting was performed according to a standard protocol (Wang J. et al., 2018; Xie et al., 2019). The primary antibodies, including for E-cadherin (Cat No. 60335-1), Vimentin (Cat No. 10366-1), ZO-1 (Cat No. 66452-1), N-cadherin (Cat No. 66219-1), GAPDH (Cat No. 60004-1), and β-actin (Cat No. 66009-1), were obtained from Proteintech (Rosemont, IL, United States). The TAp63α antibody (Cat No. TA802078) was purchased from OriGene Technologies (Rockville, MD, United States). The secondary anti-mouse antibody (Cat No. L3032-2), and anti-rabbit (Cat No. L3012-2) antibodies were purchased from Signalway (Beijing, China).

### Tissue Immunohistochemical Staining

According to the manufacturer’s protocol, immunohistochemical staining was performed using the Vectastain Elite ABC Kit (Vector Laboratories, Burlingame, CA, United States). Briefly, paraffin-embedded lung cancer tissues and the adjacent tissues were deparaffinized and hydrated in xylene, ethanol, and water. After heat-induced antigen retrieval procedures, the sections were incubated overnight at 4°C with primary antibodies, including TAp63α (dilution 1:200), E-cadherin (dilution 1:500), and N-cadherin (dilution 1:500). After the primary antibodies were washed off, the ABC detection system was used together with biotinylated anti-rabbit IgG or biotinylated anti-mouse IgG. The slides were counterstained with hematoxylin and mounted in xylene mounting medium for examination. The tissue cores of the H&E-stained slices were punched (1.5 mm in diameter) in the selected tissue areas and placed on a recipient block.

### Transwell Assay

The cell migration and invasion capabilities were measured using transwell inserts (BD bioscience, SanJose, CA) using an 8 μm filter. A total of 2 × 10^3^ H1299 cells were cultured in the upper chamber of the insert in a serum-free medium. Complete medium was added to the lower chamber. Subsequently, methanol was used to fix the indicated cells, which were stained using Giemsa. Then, the cells on the top surface of the membrane were wiped off, and the cells on the lower surface were visualized and photographed using a microscope. In addition, 50 μL of Matrigel (BD Biosciences, Franklin Lakes, NJ, United States) was added to the upper chambers for the invasion assay.

### Transition Transfection

Cells were transiently transfected with pcMV-TAp63α plasmids (2 μg) or a control vector (2 μg), TAp63α-siRNA (75 nM) or control siRNA (75 nM), miR-19a/miR-19b mimics (50 nM), and miR-19a/miR-19b inhibitors (50 nM), according to the manufacturer’s protocols, using Lipofectamine 2000 (Invitrogen). miR-19a/miR-19b mimics and miR-19a/miR-19b inhibitors were purchased from RiboBio (Guangzhou, China). The pcMV-TAp63α (catalog # RG208013) was purchased from OriGene (Rockville, MD, United States). Invitrogen synthesized TAp63α-specific siRNAs and the control siRNA.

### Dual-Luciferase Reporter Assay

The wild-type (wt)-miR-17-92 promoter and mutant (mut)-miR-17-92 promoter were cloned into the pGL3 vector via directional cloning. H1299 cells were co-transfected with 0.2 μg of firefly luciferase reporter vector, 0.02 μg of the control vector containing *Renilla* luciferase, pRL-SV40, TAp63α, and TAp63α-siRNA using Lip 2000 (Invitrogen, Carlsbad, CA) in 24-well plates. Luciferase assays were performed 48 h after transfection. Firefly luciferase activity was normalized to that of *Renilla* luciferase. Luciferase reporter gene plasmids of the wt-miR-17-92 promoter and mut-miR-17-92 promoter were obtained from GENEray (Shanghai, China). pRL-SV40 plasmids were obtained from Promega (Madison, WI, United States).

### Statistics

Statistical analyses were performed using SPSS 25.0 software (SPSS, Chicago, IL, United States). Data are expressed as the mean ± standard deviation. One-way ANOVA was used to compare the statistical differences between multiple groups, and a *t*-test was used for comparisons between two groups. Differences with a *p* < 0.05 were considered statistically significant.

## Results

### TAp63α Is Correlated With Lung Cancer EMT

**FIGURE 1:**
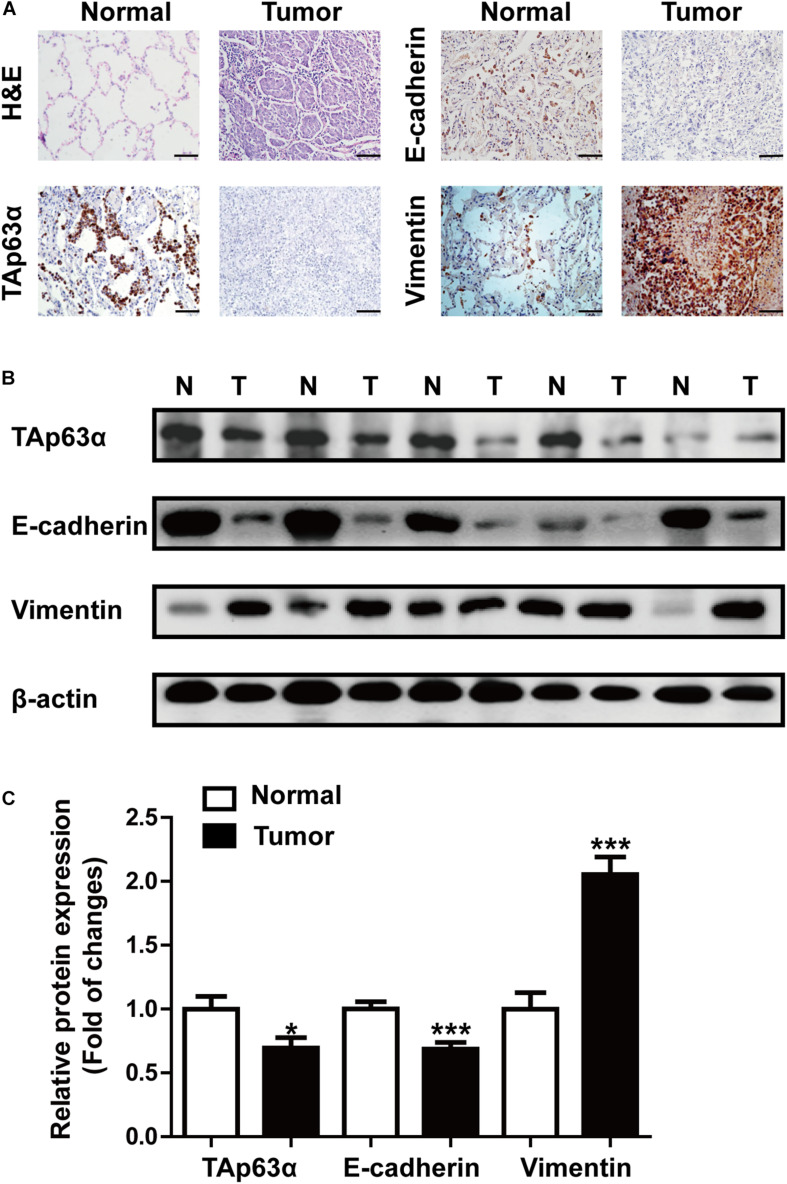
TAp63α is correlated with lung cancer EMT. **(A)** Immunohistochemistry analysis (200×) was used to determine TAp63α, E-cadherin, and N-cadherin protein levels in lung cancer tissues and normal pneumocyte tissues. Scale bar, 100 μm. **(B)** The expression of EMT-related markers, including E-cadherin and Vimentin was measured using western blotting. **(C)** Densitometry results are shown as fold change compared with the control group after normalization to β-actin (*n* = 32). Results are shown as mean ± SD. *P < 0.05, ***P < 0.001 compared with the normal group.

We first measured the expression of TAp63α in lung tissues collected from lung cancer patients using immunohistochemical (IHC) staining and western blotting, respectively. IHC results showed that lung cancer tissues had a dramatically reduced expression of TAp63α compared with the normal tissues (Figure 1A). Interestingly, decreased E-cadherin expression and increased Vimentin expression were also observed in lung cancer tissues (Figure 1A). In line with the IHC assay data, western blot analysis confirmed the decreased expression levels of TAp63α and E-cadherin and the increased Vimentin expression level in lung cancer tissues compared to the corresponding relative normal tissues (Figures 1B,C). These results indicate that TAp63α might be correlated with the TS-induced lung cancer EMT process.

### Inhibition of TAp63α Promotes Lung Cancer EMT Process

**FIGURE 2:**
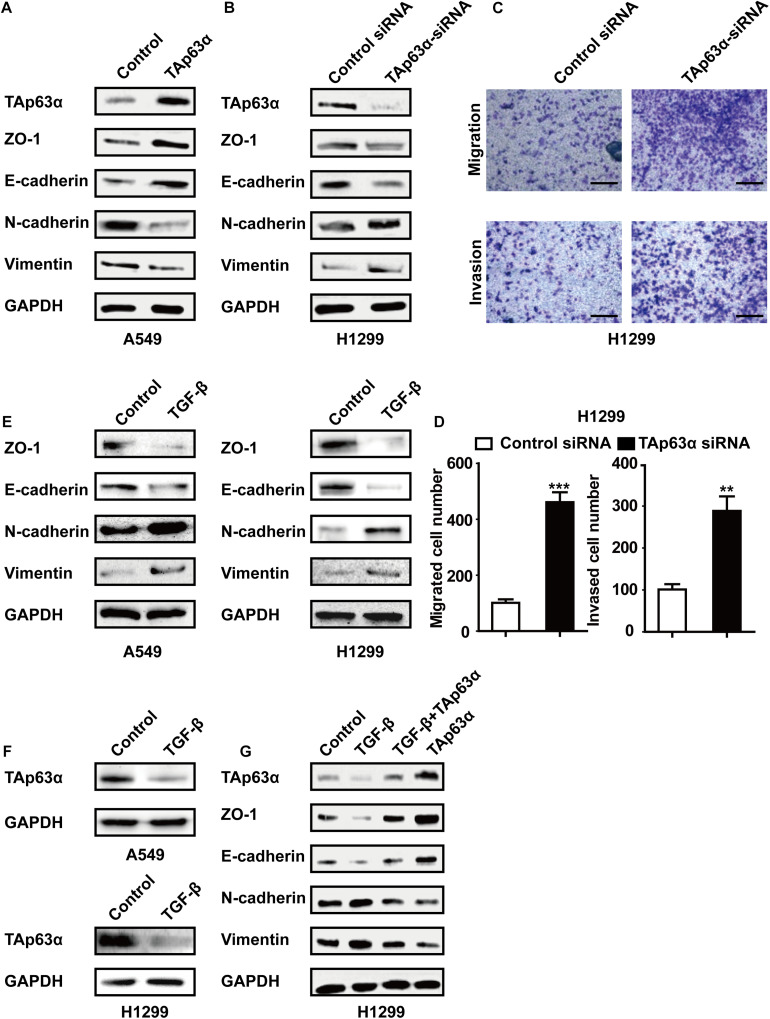
Inhibition of TAp63α promotes lung cancer EMT process. **(A)** A549 cells were treated with TAp63α transfection plasmids (2 ng), and the EMT-related biomarkers were analyzed using western blotting. **(B)** H1299 cells were transfected with TAp63α siRNA (75 μM) for 48 h, and the expression of the indicated proteins was measured using western blot in H1299 cells following transfection with TAp63α siRNA. **(C,D)** The cell abilities of migration and invasion were detected using a Transwell assay and the number of migrated and invaded H1299 cells was determined (100 ×). **(E,F)** A549 and H1299 cells were treated with TGF-β (2 ng/mL) for 2 days, western blotting analysis was used to measure the levels of TAp63α, ZO-1, E-cadherin, N-cadherin, and Vimentin. **(G)** H1299 cells were exposed to TGF-β in the presence or absence of TAp63α plasmids (2 ng) for 2 days, and the indicated gene expression was detected. Results are shown as mean ± SD of at least three independent experiments. **P < 0.01, ***P < 0.001 compared with the control group.

To explore the role of TAp63α in lung cancer EMT, A549 cells were transfected with TAp63α plasmids, and the EMT-relevant biomarkers were measured using western blotting. Figure 2A showed that the overexpression of TAp63α elevated the levels of ZO-1 and E-cadherin, but decreased N-cadherin and Vimentin expression levels. In contrast, in response to TAp63α-siRNA transfection, the protein expression levels of E-cadherin and ZO-1 were decreased, while those of N-cadherin and Vimentin were increased in these cells (Figure 2B). The suppression of TAp63α significantly increased the number of migrated and invaded H1299 cells (Figures 2C,D), suggesting that TAp63α might act as a tumor suppressor gene in lung cancer progression. To further verify the regulation of TAp63α on EMT in lung cancer, 2 ng/mL TGF-β (a classical EMT inducer) was used to trigger the lung cancer EMT process, and the relevant gene expression was analyzed using western blot analysis. As expected, TGF-β remarkably altered the expression of EMT-associated biomarkers, including decreased ZO-1 and E-cadherin expression and increased N-cadherin and Vimentin expression in A549 and H1299 cells (Figure 2E). The level of TAp63α was also reduced in TGF-β-treated lung cancer cells (Figure 2F). Interestingly, TAp63α overexpression restored TGF-β-induced alterations in EMT-associated biomarkers in H1299 cells (Figure 2G). The above data suggest that the suppression of TAp63α promotes the lung cancer EMT process.

### TAp63α Affects the TS-Induced Lung Cancer EMT Process

**FIGURE 3:**
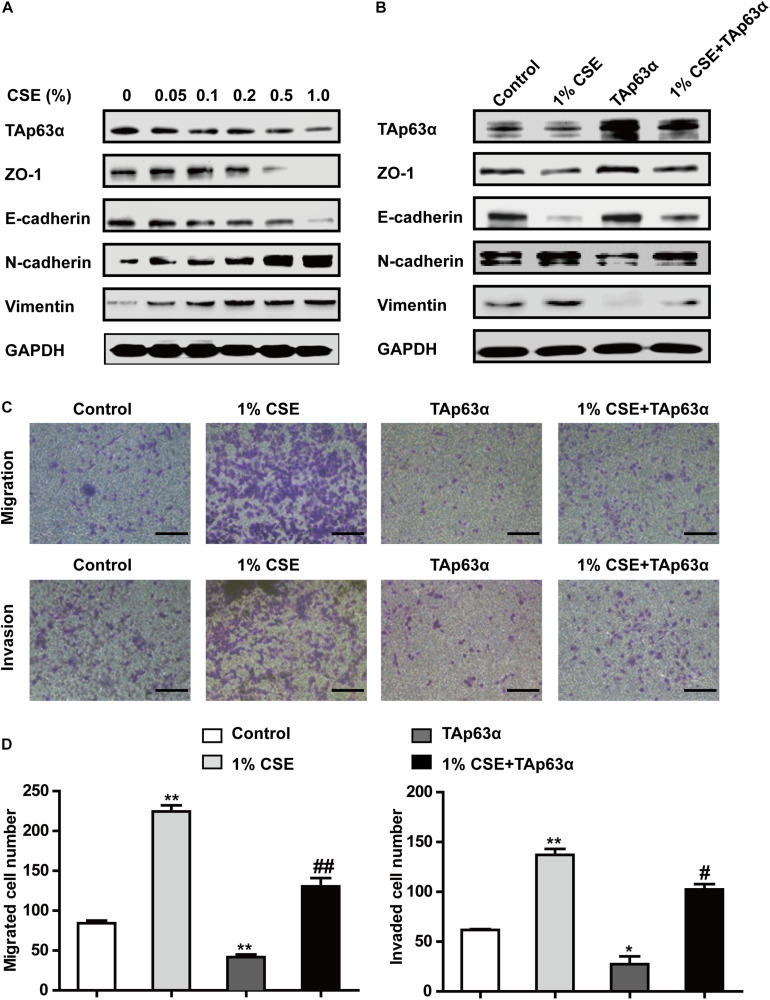
TAp63α affects the TS-induced lung cancer EMT process. **(A)** H1299 cells were treated with various concentrations of CSE (0–1%) for 2 days, and the expression of TAp63α and EMT-associated markers was analyzed using western blotting. **(B)** H1299 cells were treated with 1% CSE in the presence or absence of TAp63α plasmid transfection for 48 h, and western blotting analysis was used to detect the expression of the indicated proteins. **(C)** The cell migration and invasion abilities were tested using Transwell assays (100×). **(D)** Migrated and invaded cell numbers. Results are shown as the mean ± SD of at least three independent experiments. *P < 0.05, **P < 0.01, compared with the control group. ^#^*P* < 0.05, ^##^*P* < 0.01, compared with the 1% CSE treatment group. CSE: cigarette smoke extract.

To further investigate the role of TAp63α in TS-induced lung cancer EMT, H1299 cells were treated with various concentrations of CSE for 48 h, and the expression of EMT markers was determined using western blotting. As shown in Figure 3A, CSE dose-dependently decreased the expression of ZO-1 and E-cadherin and increased that of N-cadherin and Vimentin. As expected, TAp63α expression level was reduced in CSE-exposed H1299 cells (Figure 3A). Interestingly, the overexpression of TAp63α blocked CSE-stimulated alterations in EMT-associated biomarkers (Figure 3B). CSE exposure enhanced the migration and invasion abilities of H1299 cells (Figures 3C,D), which were significantly reversed in H1299 cells overexpressing TAp63α (Figures 3C,D). These results suggest that TAp63α is involved in TS-induced lung cancer EMT.

### TAp63α Transcriptionally Suppresses miR-19 to Inhibit TS-Induced Lung Cancer EMT

**FIGURE 4:**
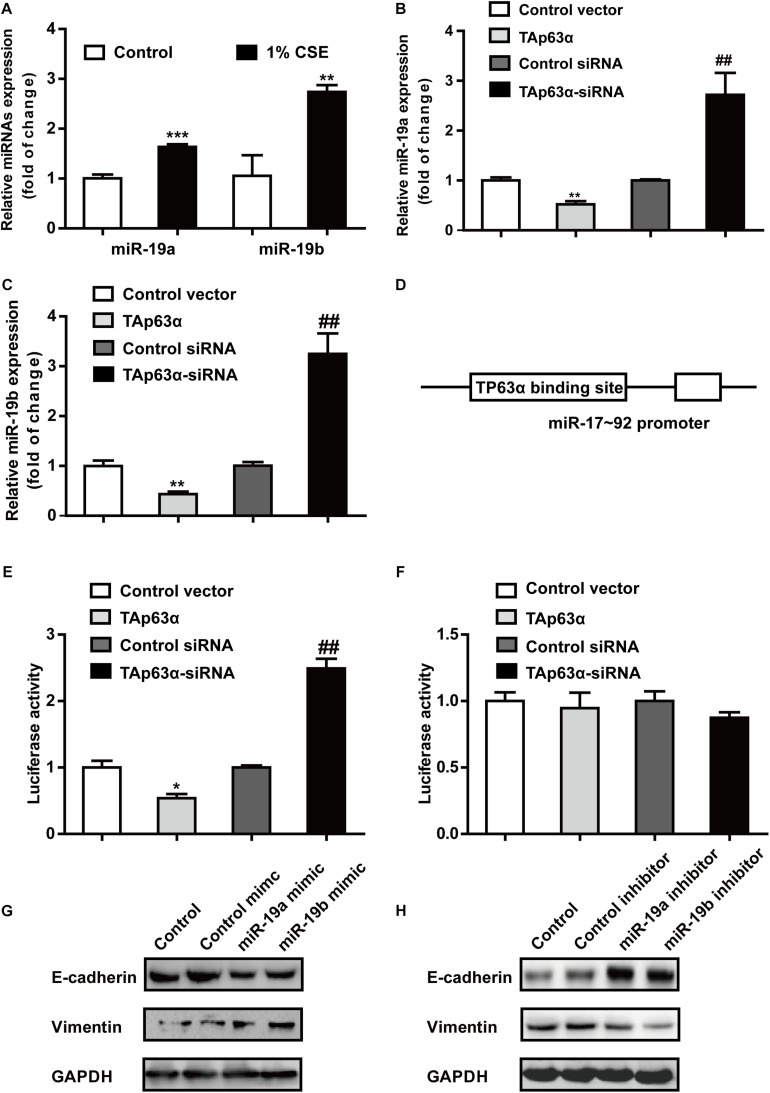
TAp63α transcriptionally suppresses miR-19 to inhibit TS-induced EMT in lung cancer. **(A)** RT-qPCR was used to detect the levels of miR-19a and miR-19b in H1299 cells exposed to 1% CSE for 2 days. **(B,C)** H1299 cells were transfected with TAp63α siRNA (75 μM) or TAp63α plasmids (2 ng) for 48 h, and RT-qPCR was used to detect the expression of miR-19a and miR-19b in H1299 cells. **(D)** Bioinformatics analysis of the binding site of the miR-17-92 promoter and TP63α. **(E)** H1299 cells were transfected with wild-type miR-17-92 promoter (wt-miR-17-92 promoter), TAp63α plasmids, or TAp63α siRNA, and the luciferase activity was measured. **(F)** A dual-luciferase reporter assay was used to detect the luciferase activity in H1299 cells transfected with mutant type miR-17-92 promoter (mut-miR-17-92 promoter) and TAp63α plasmids or TAp63α siRNA. **(G,H)** The expression of E-cadherin and Vimentin was analyzed in miR-19 overexpression and miR-19 knockdown H1299 cells. Results are shown as the mean ± SD of at least three independent experiments. *P < 0.05, **P < 0.01, ***P < 0.001, compared with the control group. ^##^*P* < 0.01, compared with the control siRNA group.

miR-19 acts as an oncogene that promotes lung cancer progression. RT-qPCR results revealed that 1% CSE dramatically elevated miR-19a and miR-19b expression in H1299 cells (Figure 4A). To investigate the potential role of miR-19 in regulating TAp63α in lung cancer EMT, H1299 cells were transfected with TAp63α plasmids and TAp63α siRNA, and the levels of miR-19a and miR-19b were measured using qRT-PCR. As shown in Figures 4B,C, TAp63α overexpression significantly reduced the expression of miR-19a and miR-19b, while TAp63α knockdown increased miR-19a and miR-19b expression levels in H1299 cells, suggesting the regulation of miR-19 by TAp63α. To further analyze the binding site of TP63α onto the miR-17-92 promoter, bioinformatics analysis was performed. As shown in Figure 4D, there was a TP63α binding site in the response element (TP63αRE) of the miR-17-92 promoter. A dual-luciferase reporter assay using luciferase plasmids of miR-17-92 p63αRE was performed to detect the transcriptional activity of miR-19. As shown in Figure 4E, TAp63α overexpression significantly reduced the luciferase activity of the miR-17-92 promoter, while TAp63α siRNA promoted the luciferase activity of the miR-17-92 promoter in H1299 cells. In addition, after mutating the TP63α binding site in the miR-17-92 promoter, the luciferase reporter assay showed that the overexpression or knockdown of TAp63α failed to induce alterations in the fluorescence intensity of the mutated miR-17-92 promoter (Figure 4F). Furthermore, the function of miR-19 in EMT in lung cancer was explored. As presented in Figure 4G, the upregulation of miR-19a and miR-19b by transfecting miR-19a/miR-19b mimic increased Vimentin expression and decreased E-cadherin expression. The inhibition of miR-19a and miR-19b reduced the level of Vimentin and elevated the level of E-cadherin in H1299 cells (Figure 4H). Together, these data indicate that TAp63α transcriptionally suppresses miR-19 expression in lung cancer cells.

### Curcumin Blocks the TS-Triggered Lung Cancer EMT Process by Targeting TAp63α and miR-19

**FIGURE 5:**
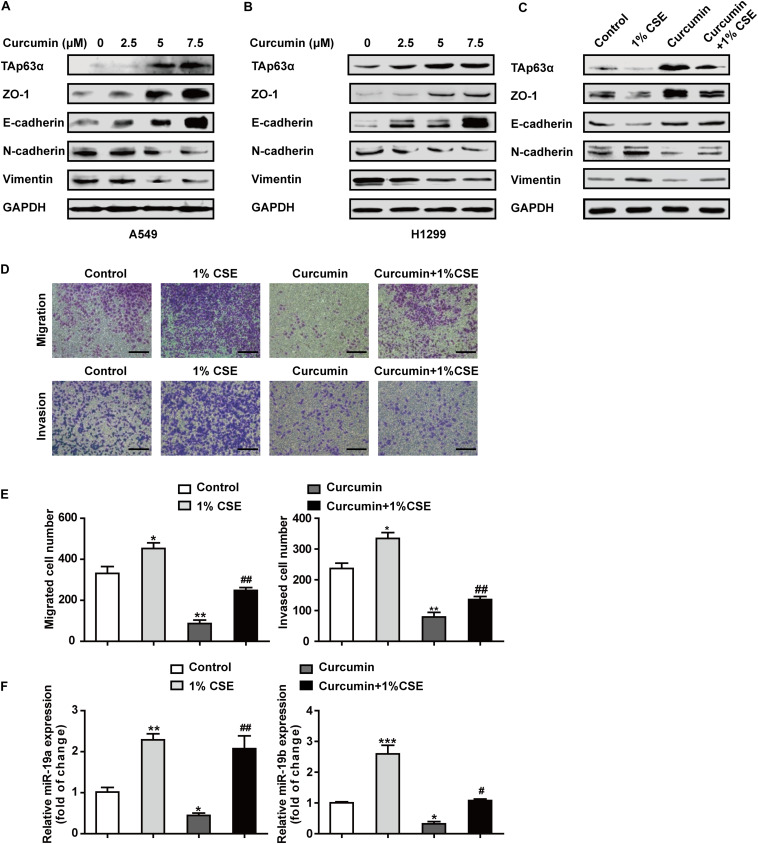
Curcumin blocks the TS-triggered lung cancer EMT process by targeting TAp63α and miR-19. **(A,B)** A549 and H1299 cells were exposed to various concentrations (0, 2.5, 5, and 7.5 μM) of curcumin for 2 days, and the levels of TAp63α and EMT-related markers, including ZO-1, E-cadherin, N-cadherin, and Vimentin were detected using western blot. **(C)** H1299 cells were co-treated with curcumin (7.5 μM) and 1% CSE for 2 days, and the indicated gene expression was analyzed. **(D)** A Transwell assay was used to measure the cell migration and invasion capacities (100×). **(E)** The migrated and invaded cell numbers. **(F)** RT-qPCR was used to detect the levels of miR-19a and miR-19b in those cells. Results are shown as mean ± SD of at least three independent experiments. *P < 0.05, **P < 0.01, ***P < 0.001, compared with the control group. ^#^*P* < 0.05, ^##^*P* < 0.01, compared with the 1% CSE treatment group. CSE: cigarette smoke extract.

To understand the chemopreventive action of curcumin on lung cancer EMT, A549, and H1299 cells were exposed to various concentrations of curcumin (0, 2.5, 5, and 7.5 μM) for 48 h, and the protein expression of EMT-associated biomarkers was measured using western blot. As shown in Figures 5A,B, curcumin increased the expression of TAp63α, along with ZO-1 and E-cadherin expression, in a concentration-dependent manner. The levels of N-cadherin and Vimentin were reduced in A549 and H1299 cells, following curcumin treatment (Figures 5A,B). To further explore whether curcumin could inhibit TS-induced lung cancer EMT, H1299 cells were treated with curcumin in the presence or absence of 1% CSE, and the protein expression of EMT-relevant biomarkers was analyzed. Western blot results revealed that the levels of TAp63α, ZO-1, and E-cadherin were increased in H1299 cells exposed to co-treatment with curcumin and 1% CSE compared with the 1% CSE treatment group (Figure 5C). Curcumin also restored the N-cadherin and Vimentin expression levels in H1299 cells exposed to 1% CSE (Figure 5C). Moreover, curcumin significantly decreased the CSE-induced elevation of the migrated and invaded cell numbers (Figures 5D,E). Additionally, the gene expression of miR-19 was detected in H1299 cells co-treated with 1% CSE and curcumin. qRT-PCR results showed that curcumin alleviated the TS-induced upregulation of miR-19a and miR-19b in H1299 cells (Figure 5F). These data indicate that curcumin protects against TS-triggered lung cancer EMT, possibly via TAp63α and miR-19.

## Discussion

**FIGURE 6:**
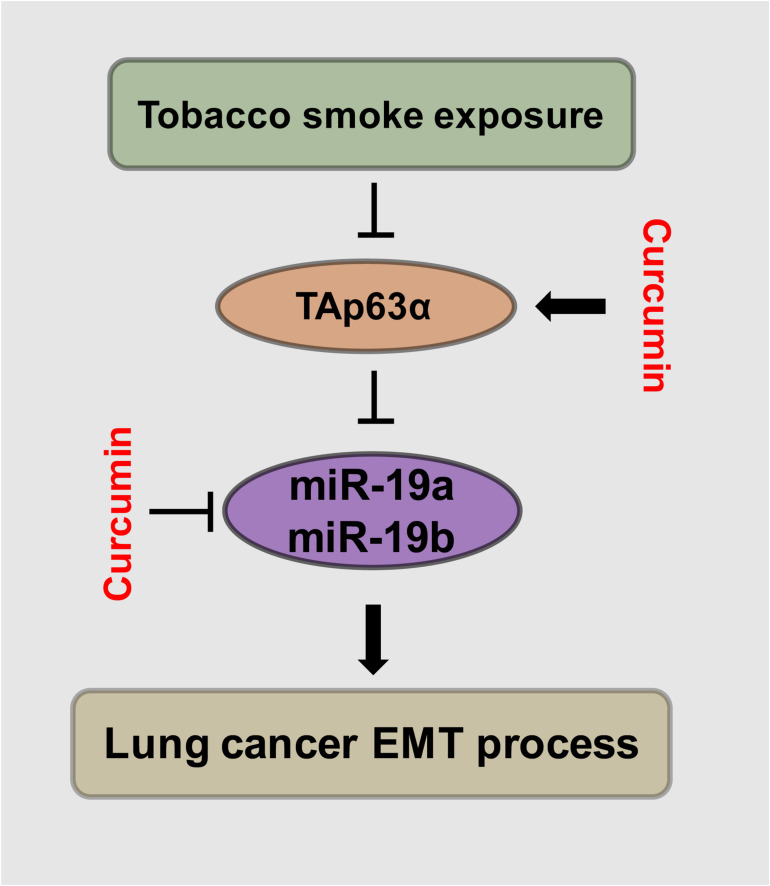
Schematic illustration of the regulatory effect of TAp63α on TS-induced lung cancer EMT and the curcumin anti-cancer effects. TGF-β treatment and tobacco smoke exposure decreased TAp63α expression; TAp63α transcriptionally reduces the expression of miR-19a/miR-19b, thereby inhibiting the lung cancer cell EMT process. Additionally, curcumin alleviates TS-stimulated lung cancer EMT via TAp63α upregulation and a miR-19 level decrease.

TAp63α is a critical transcription factor. TS plays an essential role in lung cancer development. Curcumin exhibits anti-cancer effects in various types of solid tumors. This study was designed to investigate the role and potential mechanism of TAp63α in TS-induced lung cancer EMT and the preventive effects of curcumin. We found that TAp63α inhibits TS-induced lung cancer EMT via transcriptional suppression of miR-19. Curcumin alleviated TS-stimulated lung cancer EMT by targeting TAp63α and miR-19 (Figure 6).

TS exposure is closely related to lung cancer initiation and development. The long-term exposure to TS induces persistent inflammation, oncogenic activation, and inactivation of tumor suppressor genes, leading to cell proliferation, EMT, migration, and invasion (Martey et al., 2004; Macowan et al., 2020). Numerous studies have focused on the modulation of oncogenes in TS-induced lung cancer progression (Jhanwar et al., 2020). However, few reports have identified the mechanism of modulation of tumor suppressor genes in lung cancer. In the present study, we showed that TS decreased TAp63α expression in lung cancer cells undergoing EMT. The overexpression of TAp63α alleviated TS-induced lung cancer EMT. These data indicate that TAp63α acts as a tumor suppressor gene and might mediate TS-triggered lung cancer EMT.

As an essential transcription factor, TAp63α regulates a wide range of cellular processes during tumor development. However, the function of TAp63α in tumor progression remains controversial. TAp63α increases the cell adhesion ability and correlates with patient survival in cervical squamous cell carcinoma (Nekulova et al., 2013; Masuda et al., 2015). Conversely, several lines of evidence have suggested a differential regulatory role of TAp63α in cancer development. TAp63α suppresses cellular metastasis in colon cancer (Lin et al., 2014) and inhibits mammary tumorigenesis (Su et al., 2017). The downregulation of TAp63 enhanced EMT in MDCK cells (Zhang et al., 2014). Thus, the above studies have illustrated that TAp63α regulation of tumor progression may depend on tissue and/or cell types. In the present study, a lower level of TAp63α was observed in lung cancer tissues. The suppression of TAp63α altered the expression of EMT-related biomarkers. The classical tumor EMT inducer TGF-β further confirmed that TAp63α acts as a tumor suppressor gene in lung cancer development and the inhibition of TAp63α triggered the EMT process in lung cancer.

Emerging evidence has demonstrated that TAp63α transcriptionally regulates multiple miRNAs to mediate migration and tumor growth (Su et al., 2010; Lin et al., 2014). miRNAs exhibit their ability to modulate almost all tumor-associated processes, including cancer EMT. Previous studies have reported that miR-19, which is a critical oncogenic component of the miR-17–92 cluster, promotes EMT, migration, and invasion of lung cancer cells (Li et al., 2015). The upregulation of miR-19 is positively correlated with a poor survival in lung cancer (Lin et al., 2013). However, the possible molecular mechanisms of miR-19 in lung cancer development have yet to be investigated. In the present study, luciferase reporter assays and qRT-PCR analysis showed that TAp63α transcriptionally suppressed the level of miR-19 in lung cancer cells. Moreover, the inhibition of TAp63α, along with the elevation of miR-19, was also observed in lung cancer cells upon TS exposure. These data indicate that the TS-induced decrease in TAp63α expression transcriptionally enhances the level of miR-19, and finally leads to the lung cancer EMT process.

As a natural compound, curcumin possesses a high capacity to exert anti-inflammatory and anti-cancer effects by regulating various genes, such as oncogenes and transcription factors (Zhang et al., 2016; Giordano and Tommonaro, 2019; Wang et al., 2019). Curcumin suppresses cancer stem cell activity, enhances drug sensitivity, and inhibits cell growth in lung cancer cells through the Wnt/β-catenin and PI3K/AKT pathways (Wang J. Y. et al., 2018; Chen et al., 2019). In this study, we found that curcumin exhibited anti-cancer properties in TS-induced lung cancer EMT. Curcumin dose-dependently increased the expression of TAp63α in lung cancer cells. CSE-enhanced cell migration and invasion capabilities were inhibited in lung cancer cells following curcumin treatment. Moreover, curcumin inhibited the CSE-induced upregulation of miR-19a and miR-19b in lung cancer cells. The above data demonstrate that TAp63α and miR-19 might participate in the anti-cancer effect of curcumin on TS-induced lung cancer.

## Conclusion

In summary, the present study demonstrated that TAp63α acts as a tumor suppressor and transcriptionally reduces miR-19, which results in the inhibitory effect on TS-induced lung cancer EMT. Curcumin displayed cancer preventive properties in TS-stimulated lung cancer EMT by increasing the TAp63α level and decreasing the miR-19 level. These findings will provide a new strategy for the prevention of lung cancer.

## Data Availability Statement

The original contributions presented in the study are included in the article/supplementary material, further inquiries can be directed to the corresponding author/s.

## Ethics Statement

All procedures involving human tumors were approved by the Ethics Committee of Nanjing Medical University with ethical clearance application number (2016-318). The patients/participants provided their written informed consent to participate in this study.

## Author Contributions

CX, JZ, and XY performed mainly the experiments and analyzed data. CH performed *in vitro* experiments. LZ edited the manuscript. ZM supervised the study and provided the clinical specimens. XL designed and performed experiments, and wrote the manuscript. CZ supervised the study and approval of the article. All authors were involved in revision of the manuscript and approved the submitted version.

## Conflict of Interest

The authors declare that the research was conducted in the absence of any commercial or financial relationships that could be construed as a potential conflict of interest.

## Abbreviations

EMT: Epithelial-mesenchymal transition
miR: microRNA
TS: Tobacco smoke
TP63: The human p63 gene
IHC: immunohistochemical
H&E: hematoxylin and eosin
CSE: Cigarette smoke extract
TP63 α RE: TP63 α the response element
NSCLC: non-small cell lung cancer.

## References

[B1] Bankhead A. R. McMaster T. Wang Y. Boonstra P. S. Palmbos P. L. (2020). TP63 isoform expression is linked with distinct clinical outcomes in cancer. *Ebiomedicine* 51:102561. 10.1016/j.ebiom.2019.11.022 31927310 PMC6953644

[B2] Carroll D. K. Carroll J. S. Leong C. O. Cheng F. Brown M. Mills A. A. (2006). p63 regulates an adhesion programme and cell survival in epithelial cells. *Nat. Cell Biol.* 8 551–561. 10.1038/ncb1420 16715076

[B3] Chen P. Huang H. P. Wang Y. Jin J. Long W. G. Chen K. (2019). Curcumin overcome primary gefitinib resistance in non-small-cell lung cancer cells through inducing autophagy-related cell death. *J. Exp. Clin. Cancer Res.* 38:254. 10.1186/s13046-019-1234-8 PMC6567416 31196210

[B4] Giordano A. Tommonaro G. (2019). Curcumin and Cancer. *Nutrients* 11:2376. 10.3390/nu11102376 PMC6835707 31590362

[B5] Ingenito F. Roscigno G. Affinito A. Nuzzo S. Scognamiglio I. Quintavalle C. (2019). The role of exo-miRNAs in cancer: a focus on therapeutic and diagnostic applications. *Int. J. Mol. Sci.* 20:4687. 10.3390/ijms20194687 31546654 PMC6801421

[B6] Jhanwar S. C. Xu X. L. Elahi A. H. Abramson D. H. (2020). Cancer genomics of lung cancer including malignant mesothelioma: a brief overview of current status and future prospects. *Adv. Biol. Regul.* 78:100723. 10.1016/j.jbior.2020.100723 32992231

[B7] Ji H. Ramsey M. R. Hayes D. N. Fan C. McNamara K. Kozlowski P. (2007). LKB1 modulates lung cancer differentiation and metastasis. *Nature* 448 807–810. 17676035 10.1038/nature06030

[B8] Lemjabbar-Alaoui H. Hassan O. U. Yang Y. W. Buchanan P. (2015). Lung cancer: biology and treatment options. *Biochim. Biophys. Acta* 1856 189–210. 26297204 10.1016/j.bbcan.2015.08.002 PMC4663145

[B9] Li J. Yang S. Yan W. Yang J. Qin Y. J. Lin X. L. (2015). MicroRNA-19 triggers epithelial-mesenchymal transition of lung cancer cells accompanied by growth inhibition. *Lab. Invest.* 95 1056–1070. 10.1038/labinvest.2015.76 26098000

[B10] Li X. Fu Q. Li H. Zhu L. Chen W. Ruan T. (2019). MicroRNA-520c-3p functions as a novel tumor suppressor in lung adenocarcinoma. *FEBS J.* 286 2737–2752. 30942957 10.1111/febs.14835

[B11] Liang Z. Lu L. Mao J. Li X. Qian H. Xu W. (2017). Curcumin reversed chronic tobacco smoke exposure induced urocystic EMT and acquisition of cancer stem cells properties via Wnt/beta-catenin. *Cell Death Dis.* 8:e3066. 10.1038/cddis.2017.452 28981096 PMC5680574

[B12] Lin C. W. Li X. R. Zhang Y. Hu G. Guo Y. H. Zhou J. Y. (2014). TAp63 suppress metastasis via miR-133b in colon cancer cells. *Br. J. Cancer* 110 2310–2320. 10.1038/bjc.2014.118 24594999 PMC4007221

[B13] Lin Q. Chen T. Lin Q. Lin G. Lin J. Chen G. (2013). Serum miR-19a expression correlates with worse prognosis of patients with non-small cell lung cancer. *J. Surg. Oncol.* 107 767–771. 10.1002/jso.23312 23609137

[B14] Lin R. K. Hsieh Y. S. Lin P. Hsu H. S. Chen C. Y. Tang Y. A. (2010). The tobacco-specific carcinogen NNK induces DNA methyltransferase 1 accumulation and tumor suppressor gene hypermethylation in mice and lung cancer patients. *J. Clin. Invest.* 120 521–532. 10.1172/jci40706 20093774 PMC2810088

[B15] Lo I. M. Monica V. Saviozzi S. Ceppi P. Bracco E. Papotti M. (2011). p63 and p73 isoform expression in non-small cell lung cancer and corresponding morphological normal lung tissue. *J. Thorac. Oncol.* 6 473–481. 10.1097/jto.0b013e31820b86b0 21289519

[B16] Macowan M. G. Liu H. Keller M. D. Ween M. Hamon R. Tran H. B. (2020). Interventional low-dose azithromycin attenuates cigarette smoke-induced emphysema and lung inflammation in mice. *Physiol. Rep.* 8:e14419. 10.14814/phy2.14419 PMC7354087 32652854

[B17] Martey C. A. Pollock S. J. Turner C. K. O’Reilly K. M. Baglole C. J. Phipps R. P. (2004). Cigarette smoke induces cyclooxygenase-2 and microsomal prostaglandin E2 synthase in human lung fibroblasts: implications for lung inflammation and cancer. *Am. J. Physiol. Lung Cell. Mol. Physiol.* 287:L981-91. 10.1152/ajplung.00239.2003 15234907

[B18] Masuda Y. Takahashi H. Hatakeyama S. (2015). TRIM29 regulates the p63-mediated pathway in cervical cancer cells. *Biochim. Biophys. Acta* 1853(10 Pt A) 2296–2305. 10.1016/j.bbamcr.2015.05.035 26071105

[B19] Moll U. M. Slade N. (2004). p63 and p73: roles in development and tumor formation. *Mol. Cancer Res.* 2 371–386. 15280445

[B20] Nekulova M. Holcakova J. Nenutil R. Stratmann R. Bouchalova P. Muller P. (2013). Characterization of specific p63 and p63-N-terminal isoform antibodies and their application for immunohistochemistry. *Virchows Arch.* 463 415–425. 10.1007/s00428-013-1459-4 23887585

[B21] Olive V. Bennett M. J. Walker J. C. Ma C. Jiang I. Cordon-Cardo C. (2009). miR-19 is a key oncogenic component of mir-17-92. *Genes Dev.* 23 2839–2849. 10.1101/gad.1861409 20008935 PMC2800084

[B22] Peng X. Guan L. Gao B. (2018). miRNA-19 promotes non-small-cell lung cancer cell proliferation via inhibiting CBX7 expression. *Onco Targets Ther.* 11 8865–8874. 10.2147/ott.s181433 30584339 PMC6290863

[B23] Pillai S. Trevino J. Rawal B. Singh S. Kovacs M. Li X. (2015). Beta-arrestin-1 mediates nicotine-induced metastasis through E2F1 target genes that modulate epithelial-mesenchymal transition. *Cancer Res.* 75 1009–1020. 10.1158/0008-5472.can-14-0681 25600647 PMC4359962

[B24] Qiu F. Gu W. G. Li C. Nie S. L. Yu F. (2018). Analysis on expression level and diagnostic value of miR-19 and miR-21 in peripheral blood of patients with undifferentiated lung cancer. *Eur. Rev. Med. Pharmacol. Sci.* 22 8367–8373. 30556877 10.26355/eurrev_201801_16534

[B25] Rupaimoole R. Slack F. J. (2017). MicroRNA therapeutics: towards a new era for the management of cancer and other diseases. *Nat. Rev. Drug Discov.* 16 203–222. 10.1038/nrd.2016.246 28209991

[B26] Su X. Chakravarti D. Cho M. S. Liu L. Gi Y. J. Lin Y. L. (2010). TAp63 suppresses metastasis through coordinate regulation of Dicer and miRNAs. *Nature* 467 986–990. 10.1038/nature09459 20962848 PMC3055799

[B27] Su X. Napoli M. Abbas H. A. Venkatanarayan A. Bui N. Coarfa C. (2017). TAp63 suppresses mammary tumorigenesis through regulation of the hippo pathway. *Oncogene* 36 2377–2393. 10.1038/onc.2016.388 27869165 PMC5415945

[B28] Vu T. Jin L. Datta P. K. (2016). Effect of cigarette smoking on epithelial to mesenchymal transition (EMT) in lung cancer. *J. Clin. Med.* 5:44. 10.3390/jcm5040044 27077888 PMC4850467

[B29] Wang J. Chen J. Jiang Y. Shi Y. Zhu J. Xie C. (2018). Wnt/beta-catenin modulates chronic tobacco smoke exposure-induced acquisition of pulmonary cancer stem cell properties and diallyl trisulfide intervention. *Toxicol. Lett.* 291 70–76. 10.1016/j.toxlet.2018.04.003 29626521

[B30] Wang J. Y. Wang X. Wang X. J. Zheng B. Z. Wang Y. Wang X. (2018). Curcumin inhibits the growth via Wnt/beta-catenin pathway in non-small-cell lung cancer cells. *Eur. Rev. Med. Pharmacol. Sci.* 22 7492–7499. 30468498 10.26355/eurrev_201811_16290

[B31] Wang M. Jiang S. Zhou L. Yu F. Ding H. Li P. (2019). Potential mechanisms of action of curcumin for cancer prevention: focus on cellular signaling pathways and miRNAs. *Int. J. Biol. Sci.* 15 1200–1214. 10.7150/ijbs.33710 31223280 PMC6567807

[B32] Xie C. Zhu J. Jiang Y. Chen J. Wang X. Geng S. (2019). Sulforaphane inhibits the acquisition of tobacco smoke-induced lung cancer stem cell-like properties via the IL-6/DeltaNp63alpha/otch Axis. *Theranostics* 9 4827–4840. 10.7150/thno.33812 31367260 PMC6643434

[B33] Zhang H. Liu H. Borok Z. Davies K. J. Ursini F. Forman H. J. (2012). Cigarette smoke extract stimulates epithelial-mesenchymal transition through Src activation. *Free Radic. Biol. Med.* 52 1437–1442. 10.1016/j.freeradbiomed.2012.01.024 22342303 PMC3312989

[B34] Zhang H. Y. Yang W. Zheng F. S. Wang Y. B. Lu J. B. (2017). Long non-coding RNA SNHG1 regulates zinc finger E-box binding homeobox 1 expression by interacting with TAp63 and promotes cell metastasis and invasion in lung squamous cell carcinoma. *Biomed. Pharmacother.* 90 650–658. 10.1016/j.biopha.2017.03.104 28415044

[B35] Zhang M. Xie Y. Yan R. Shan H. Tang J. Cai Y. (2016). Curcumin ameliorates alveolar epithelial injury in a rat model of chronic obstructive pulmonary disease. *Life Sci.* 164 1–8. 10.1016/j.lfs.2016.09.001 27600540

[B36] Zhang Y. Yan W. Chen X. (2014). P63 regulates tubular formation via epithelial-to-mesenchymal transition. *Oncogene* 33 1548–1557. 10.1038/onc.2013.101 23542170 PMC4905556

